# Evaluation of antibody responses to panels of *M*. *tuberculosis* antigens as a screening tool for active tuberculosis in Uganda

**DOI:** 10.1371/journal.pone.0180122

**Published:** 2017-08-02

**Authors:** Priya B. Shete, Resmi Ravindran, Emily Chang, William Worodria, Lelia H. Chaisson, Alfred Andama, J. Lucian Davis, Paul A. Luciw, Laurence Huang, Imran H. Khan, Adithya Cattamanchi

**Affiliations:** 1 Division of Pulmonary and Critical Care Medicine, University of California- San Francisco and Zuckerberg San Francisco General Hospital, San Francisco CA United States of America; 2 Curry International Tuberculosis Center, University of California-San Francisco, San Francisco CA United States of America; 3 Center for Comparative Medicine, University of California, Davis, Davis CA United States of America; 4 Department of Medicine, Makerere University College of Health Sciences, Kampala Uganda; 5 Department of Epidemiology, Bloomberg School of Public Health, Johns Hopkins University, Baltimore MD United States of America; 6 Epidemiology of Microbial Diseases, School of Public Health, Yale University, New Haven CT United States of America; 7 Pulmonary Critical Care and Sleep Medicine Section, School of Medicine, Yale University, New Haven CT United States of America; 8 HIV, Infectious Diseases, and Global Medicine Division, University of California San Francisco and Zuckerberg San Francisco General Hospital, San Francisco CA United States of America; King's College London, UNITED KINGDOM

## Abstract

**Background:**

Improved systematic screening of high-risk groups is a key component of the tuberculosis (TB) elimination strategy endorsed by the World Health Organization (WHO). We used a multiplex microbead immunoassay to measure antibody responses to 28 *M*. *tuberculosis* (*M*.*tb*) antigens, and assessed whether combinations of antibody responses achieve accuracy thresholds required for a TB screening test.

**Methods:**

A random selection of plasma samples obtained from consecutive HIV-negative adults who were admitted to Mulago Hospital in Kampala, Uganda with cough ≥2 weeks’ but <6 months’ duration were analyzed for serological response to 28 *M*.*tb* antigens using an in-house multiplex microbead immunoassay. We compared the median difference of the antibody response to each antigen between patients with and without culture-confirmed TB, ranked each antigen according to variable importance (VIM), and assessed the sensitivity and specificity of combinations of antibody responses using an advanced classification algorithm, SuperLearner.

**Results:**

Among the 237 patients included in the analysis, 119 (50%) were female, median age was 32 years (IQR 25, 46), and 113 (48%) had TB. Median antibody levels to eight antigens were significantly different between patients with and without TB. A panel including eight of the top ranked antigens had a sensitivity of 90.6% (95% CI 89.4, 93.8) and a specificity of 88.6% (95% CI 78.2, 97.6) (Ag85B, Ag85A, Ag85C, Rv0934-P38, Rv3881, BfrB, Rv3873, and Rv2878c). With sensitivity constrained to be >90%, specificity remained close to 70% with as few as 3 antigens included in the panels.

**Conclusions:**

Measuring antibody responses to combinations of antigens could facilitate TB screening and should be further evaluated in populations being targeted for systematic screening.

## Background

In order to meet ambitious tuberculosis (TB) elimination targets, the World Health Organization (WHO) now recommends systematic screening of high-risk groups[1]. Screening for active disease has several benefits, including improved patient outcomes and reduced transmission through detection and treatment of TB at an earlier stage[2]. To facilitate screening, the WHO target product profile for a TB screening test recommends a minimum sensitivity of 90% and minimum specificity of 70% [3, 4]. These targets were selected to minimize the number of false-negative results in those with TB, and to limit the need for unnecessary and costly diagnostic testing in those without TB. In addition, the target product profile calls for a low-cost and simple-to-perform assay that could be performed by front-line health workers at community health centers[4, 5]. Unfortunately, the lack of a screening strategy that meets all of these criteria is a major challenge for uptake of the systematic screening guidelines.

Current algorithms for TB screening typically include symptoms (cough greater that 2 weeks in duration, or any TB symptom such as cough of any duration, night sweats, fevers or weight loss) and/or chest radiography[1]. The sensitivity and specificity of symptom-based screening are highly variable depending on the population being screened. For example, sensitivity is high and specificity is low in people living with HIV, but the opposite is true in people without HIV infection[6]. Chest radiography more consistently meets minimum accuracy requirements for a TB screening tests, but requires infrastructure and personnel often not found in community health centers where patients first seek care. Thus, there is an urgent need for a point-of-care screening test to rapidly and accurately screen patients for active TB[7].

Serologic tests are a promising approach to screening as they meet the non-technical requirements for a TB screening test. Serological tests are simple, do not require significant laboratory infrastructure, and have been commercialized into user-friendly platforms for a variety of diseases. However, current commercial TB serologic tests detect responses to one or at most two *Mycobacterium tuberculosis* (*M*.*tb*) antigens. These assays have limited sensitivity and specificity, and the WHO has strongly recommended against their use because of inaccuracy and imprecision while also urging additional research on potential serologic tests[8, 9]. Recently, Khan and colleagues assessed antibody responses to 28 *M*.*tb* antigens in TB patients and healthy controls in Pakistan in a multiplex microbead immunoassay using the Luminex platform (Austin, TX). They identified a panel of antibody responses to 8 *M*.*tb* antigens that had high sensitivity and specificity (90% and 80%, respectively)[10]. This multiplex assay can be performed in a user-friendly and high-throughput format. Studies in other settings and that enroll patients with a clinical suspicion of TB are now needed.

We assessed antibody responses to the same 28 *M*.*tb* antigens in stored plasma samples from a cohort of patients admitted to Mulago Hospital in Kampala, Uganda with prolonged cough (*i*.*e*., patients with presumed TB). Our objective was to determine whether one or more combinations of antibody responses to *M*.*tb* antigens could meet the minimum recommended sensitivity and specificity thresholds for a TB screening test in a high burden setting.

## Methods

### Study population

We analyzed stored plasma samples from a random selection of HIV-negative adults who were enrolled between January 2009 and May 2013 in an ongoing cohort study of patients admitted to Mulago Hospital in Kampala, Uganda with cough ≥2 weeks’ but <6 months’ duration[11–13]. As part of this parent study, each patient submitted two sputum samples for acid-fast bacilli (AFB) smear microscopy (LED fluorescence microscopy) and liquid mycobacterial culture, and one additional sample for GeneXpert MTB/RIF (Xpert) testing in accordance with standard procedures. Blood samples were also collected at the time of patient enrollment. These samples were collected in EDTA-tubes and centrifuged for 10 minutes at 2000*g*. Resulting 1mL plasma aliquots were stored at -80 degrees Celsius. For this study, patients were excluded if they had a prior history of TB treatment, if TB status could not be classified (see Outcome Definition below), or if a stored plasma sample was not available. We analyzed stored plasma samples from a random sample of eligible patients, with the number analyzed based on budgetary constraints. During data analyses, we excluded patients if serological testing showed high reactivity (Median Fluorescence Intensity [MFI] > 200) to bovine serum albumin (BSA) coated bead. All data and samples were obtained and stored according to protocols approved by the Committee on Human Research at the University of California, San Francisco, the School of Medicine Research Ethics Committee at Makerere University (Kampala Uganda), and the Uganda National Council for Science and Technology.

### Multiplex antibody assay

An in-house multiplex microbead immunoassay was used to assess the serological response to 28 *M*.*tb* antigens: Rv3881c, Rv0934 (P38), Rv2031c (HspX), Rv1860 (MPT32), Rv3804c (antigen 85a [Ag85a]), Rv1886c (Ag85b), Rv0129c (Ag85c), Rv3875 (ESAT6), Rv3874 (CFP10), Rv3841 (Bfrb1), Rv3418c (GroES), Rv2875 (MPT70), Rv1984c (CFP21), Rv1980c (MPT64), Rv0054, Rv3874-Rv3875 (CFP10-ESAT) fusion, Rv3873, Rv3619, Rv2220, Rv0831c, Rv1009, Rv1099, and Rv2032, Rv1926c, Rv2878c, Rv1677, Rv1566c, and Rv3507. These antigens were chosen based on availability of recombinant antigens, ability to discriminate well between patients with and without TB in endemic settings in previous studies, or to be useful for assessing vaccine response[14]. The assay was performed by authors (RR and IK) blinded to clinical data as described previously[10]. Briefly, the 28 recombinant *M*.*tb* antigens were expressed as polyhistidine-tagged proteins in *Escherichia coli*, purified, and coupled with carbodiimide linkages to microbead sets (Luminex Corp, Austin, TX, USA). The concentration of each antigen on the beads ranged from 5ug/ml to 100ug/ml. Optimization of antigen concentration was performed by coating different microbead sets with a range of proteins, from 5 and 100 μg/ml, for each antigen. These beads were tested against TB patient sera, which were positive for antibodies to the relevant antigen, and against sera from healthy individuals. Beads that provided the strongest specific signal for each antigen against the positive sera were selected for use in the assay. PE-anti-Human IgG was used to measure IgG antibodies in plasma/serum. The selected microbead sets were then incubated with plasma samples from study participants using the Luminex platform at the Center for Comparative Medicine Laboratory, University of California, Davis[10]. The background MFI value for the BSA-coated, negative control bead set was subtracted from the raw MFI values for each antigen-coated bead set, and the background-corrected MFI values were used in subsequent analyses.

### Outcome definition

We classified patients as having pulmonary TB if sputum mycobacterial culture results were positive for *M*.*tb*, or if culture results were negative but both AFB smear and Xpert MTB/RIF results were positive. We classified patients as not having pulmonary TB if all microbiologic testing for TB, including at least two liquid culture results, were negative and patients either had clinical improvement without anti-TB therapy or an alternate diagnosis established at the 2-month follow-up visit.

### Statistical analysis

We compared the median difference in antibody response between TB and non-TB patients using the Mann-Whitney test. The remainder of the analysis was performed using SuperLearner, an open-source machine-learning ensemble method that compares different learners (prediction methods) using cross-validated risk[15]. It then builds a SuperLearner that is a weighted linear combination of the different learners included, favoring learners that minimize mean squared error, to return predictions on the dataset using cross-validation to prevent over-fitting. For this analysis, we included logistic regression, Bayes’ generalized linear models[16], lasso[17], random forest[18], and a null model (in order to assess relative performance with other methods) as candidate learners. We used a random two-thirds of the dataset to build the SuperLearner and the full dataset with 10-fold cross-validation to make predictions. With these specifications, we first ranked the relative importance of the antibody response to each of the 28 *M*.*tb* antigens in differentiating between TB and non-TB patients by estimating the Variable Importance Measure (VIM) as described by Hubbard *et al[19]*. We then calculated sensitivity, specificity, and cross-validated area under the curve (AUC) of panels consisting of the top-ranked antigens. We constrained sensitivity to be 90% or higher as recommended for a TB screening test[4]. To assess the potential for overfitting, we performed a secondary analysis that excluded the two-thirds of the dataset used to build the SuperLearner when making predictions. In addition, we compared the mean squared error of the SuperLearner and each of its component learners using 10-fold cross-validation. All analyses were performed using R, version 3.0.2 (R Foundation, Vienna, Austria).

## Results

**Fig 1:**
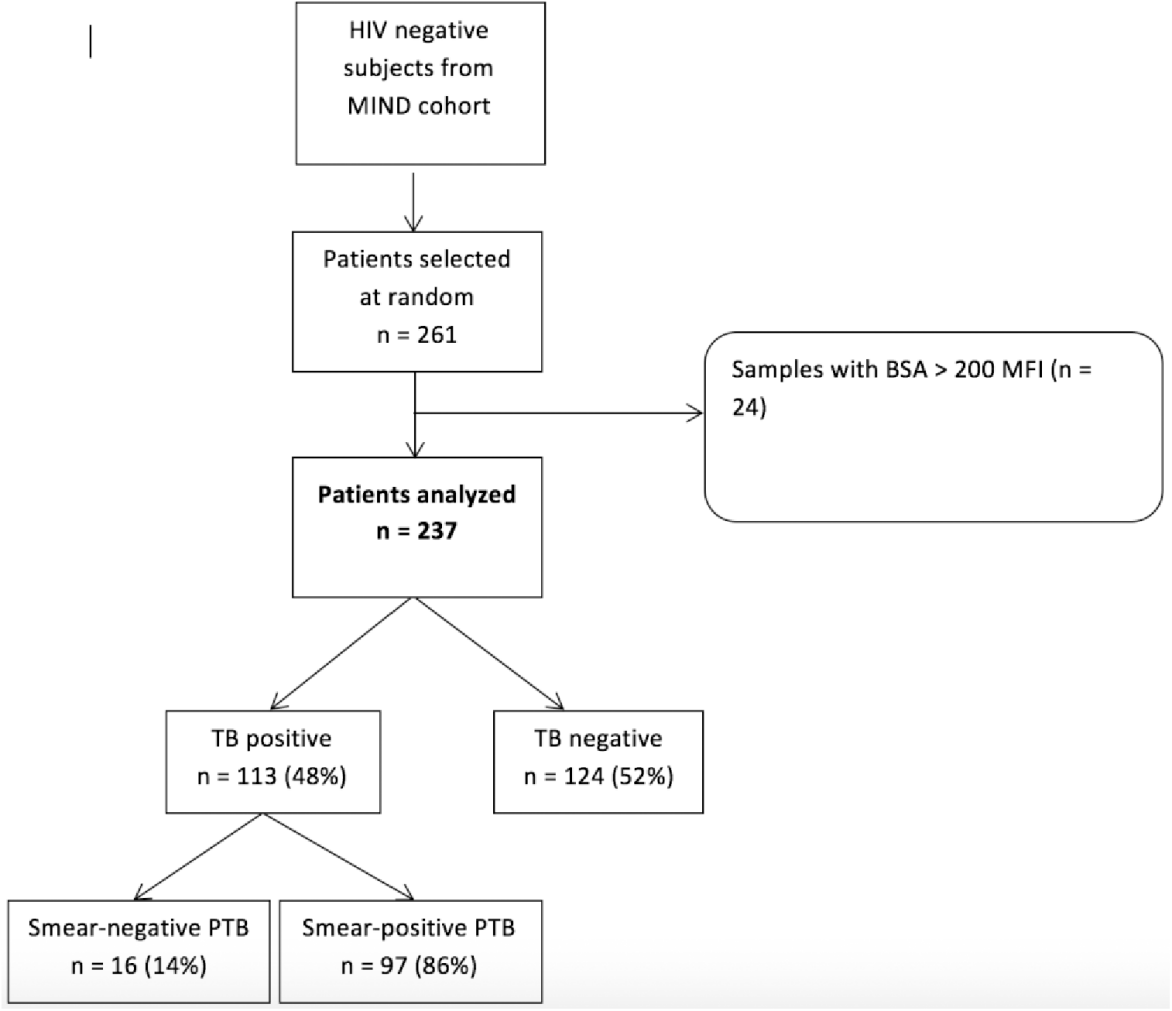
Flowchart of sample selection.

Of 777 HIV-negative patients enrolled into the parent study, 556 met eligibility criteria and 261 were randomly selected for analysis based on the available budget for antibody response testing **(Fig 1)**. Of the 261 patients selected, 24 (9.6%) were excluded for high response to the negative BSA control bead set. Of the 237 patients remaining, 119 (50%) were female, median age was 32 years (IQR 25, 46), and 113 (48%) had TB. Of those patients diagnosed with TB, 97 (86%) were sputum smear positive and 16 (14%) were sputum smear negative.

### Ranking of antigens

**Fig 2:**
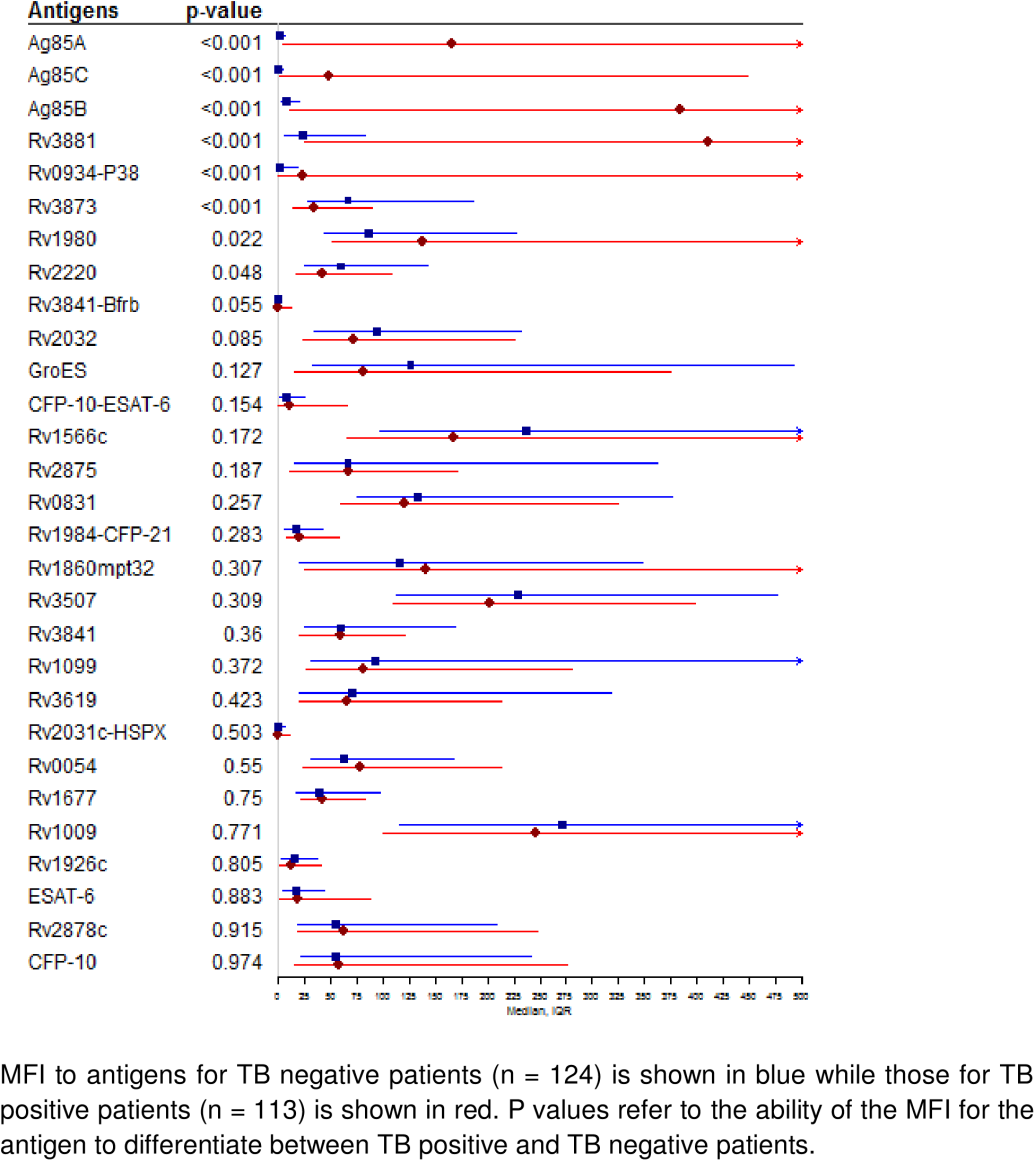
Median serological response (MFI) to 28 recombinant *M*. *tuberculosis* antigens.

Median antibody levels to eight antigens (Ag85A, Ag85C, Ag85B, Rv3881, Rv0934-P38, Rv3873, Rv1980, Rv2220) were significantly different between patients with and without TB **(Fig 2)**. Median antibody levels were >10-fold higher in TB patients for Ag85A, Ag85C, Ag85B, Rv3881, and Rv0934-P38; approximately 1.5-fold higher in TB patients for Rv1980; and approximately 1.5-fold lower in TB patients for Rv3873 and Rv2220.

Results were similar when antigens were considered together and ranked by variable importance using SuperLearner **(Table 1)**. The 5 top-ranked antigens based on variable importance (Ag85B, Ag85A, Ag85C, Rv0934-P38, Rv3881) were also the 5 top-ranked based on median fold difference (Ag85A, Ag85C, Ag85B, Rv3881, Rv0934-P38), though their rank order was different. Two antigens, BfRB and Rv2878c, were ranked high by variable importance even though median levels were similar in TB vs. Non-TB patients. In contrast, two antigens, Rv1980 and Rv2220, were ranked low by variable importance even though median levels were significantly different in TB vs. Non-TB patients. Of note, ESAT-6 and CFP-10, two antigens used in commercial interferon-gamma release assays to identify *M*.*tb* infection, were not ranked highly based on either median difference or variable importance.

**Table 1:** Ranking of 28 recombinant *M*. *tuberculosis* antigens based on variable importance (VIM).

Antigen	VIM	95% CI	p-value*
Ag85B	0.076	[0.013, 0.18]	< .01
Ag85A	0.028	[0.005, 0.082]	< .01
Ag85C	0.015	[0.001, 0.046]	0.01
Rv0934-P38	0.018	[0.001, 0.061]	0.02
Rv3881	0.013	[0.002, 0.03]	0.04
Rv3841-BfrB	0.033	[-0.018, 0.125]	0.05
Rv3873	-0.022	[-0.057, -0.001]	0.06
Rv2878c	-0.009	[-0.03, 0.011]	0.16
Rv3507	0.016	[-0.014, 0.06]	0.35
ESAT-6	-0.006	[-0.034, 0.019]	0.4
CFP-10	-0.006	[-0.03, 0.018]	0.41
CFP-10-ESAT-6	0.003	[-0.018, 0.024]	0.48
Rv1009	-0.007	[-0.033, 0.01]	0.48
Rv3619	-0.006	[-0.028, 0.018]	0.52
Rv1566c	-0.007	[-0.042, 0.022]	0.56
Rv2032	-0.007	[-0.035, 0.019]	0.64
Rv0054	0.003	[-0.011, 0.023]	0.65
Rv2875	-0.004	[-0.045, 0.031]	0.71
Rv2220	-0.004	[-0.041, 0.04]	0.72
Rv0831	-0.003	[-0.028, 0.023]	0.73
Rv1926c	-0.005	[-0.036, 0.032]	0.73
Rv1677	0.004	[-0.02, 0.037]	0.74
Rv1099	0.003	[-0.022, 0.033]	0.74
Rv1984-CFP-21	0.003	[-0.015, 0.023]	0.8
Rv1980	-0.002	[-0.021, 0.016]	0.82
Rv1860-mpt32	-0.001	[-0.018, 0.021]	0.9
GroES	0.001	[-0.025, 0.028]	0.92
Rv2031c-HSPX	0.000	[-0.015, 0.021]	0.97

* p-value refers to comparison between VIM ranking and ability of this antigen to differentiate between TB and non-TB groups

### Diagnostic accuracy of antigen panels

**Fig 3:**
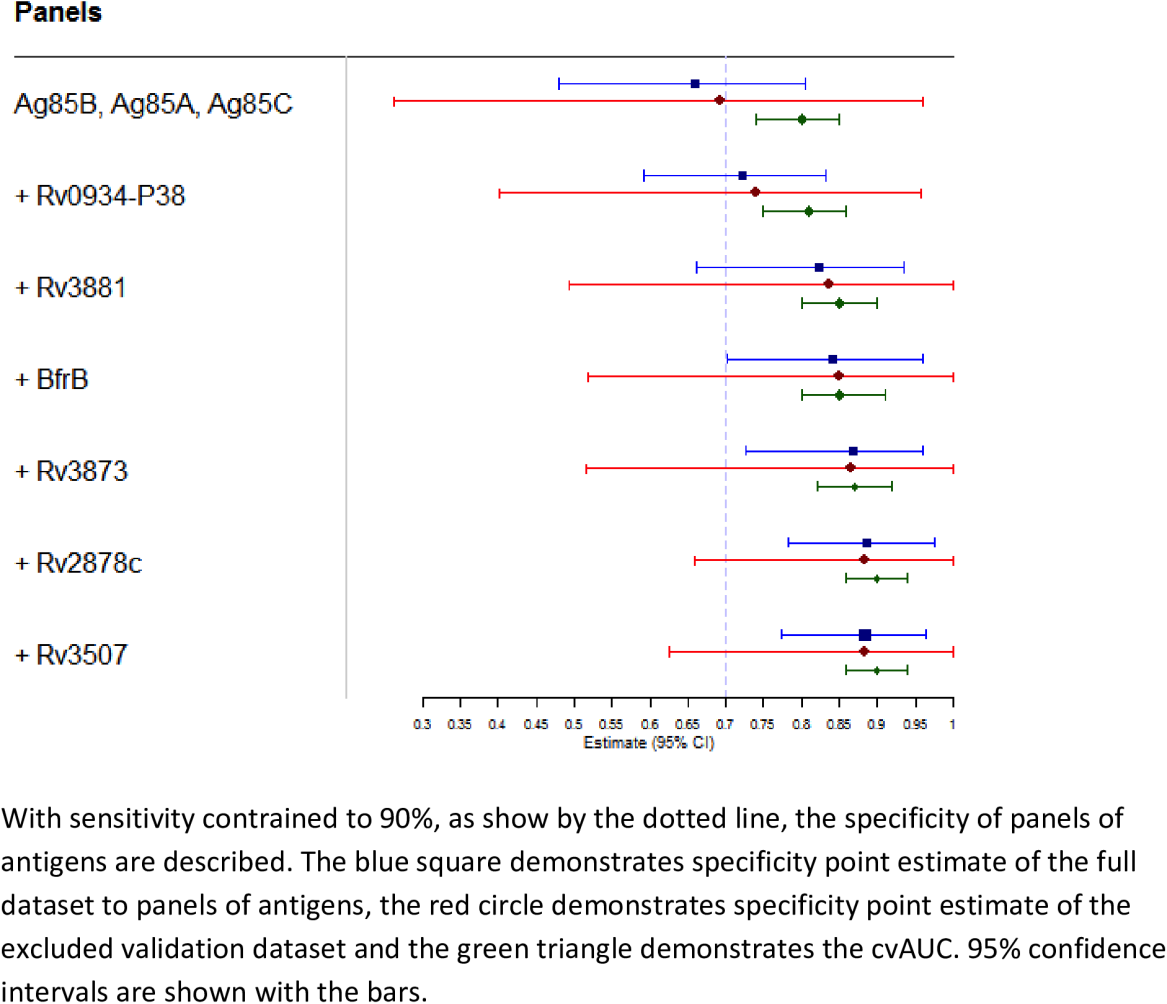
Specificity of panels consisting of top-ranked antigens in both full and excluded validation datasets.

A panel consisting of the three top-ranked antigens (Ag85A, Ag85C, Ag85B) based on variable importance had a sensitivity of 90% and specificity of 66%. With sensitivity constrained to 90%, specificity increased to 72.2%, 82.3%, 84.1%, 86.8%, and 88.6% when the 4^th^ through 8^th^ ranked antigens were added to the panel, respectively **(Fig 3, S1 Table)**. No further improvements in specificity or overall accuracy were achieved by including more than 8 antigens in the panel. Data were similar when predictions were made using only the validation dataset (*i*.*e*., one-third of the data not used to fit SuperLearner) **(Fig 3, S1 Table)**. With sensitivity constrained to 90%, specificity ranged from 69.2% to 88.4% for the same 3- to 8-antigen panels, with specificity increasing as panel size increased. The SuperLearner performed as well as the best individual learner in its library **(S2 Table)**, as determined by ability to minimize estimated mean squared error.

## Discussion

A simple, low-cost and accurate biomarker-based screening test for active TB is among the highest priorities for TB diagnostics. In this study, we analyzed antibody responses to 28 *M*.*tb* antigens and identified at least eight (Ag85B, Ag85A, Ag85C, Rv0934-P38, Rv3881, BfrB, Rv3873, and Rv2878c) that showed potential for utility in TB screening. These eight antigens were the top-ranked antigens based on variable importance and five of them also demonstrated median levels that were >10-fold higher in TB patients than in patients without TB. With sensitivity constrained to ≥90%, specificity was approximately 70% for a panel consisting of the 3 top-ranked antigens and increased to approximately 90% for a panel consisting of all 8 top-ranked antigens. Thus, the antigen panels identified here meet or exceed the minimum accuracy targets for a TB screening test[3, 4] and should be further evaluated in populations targeted for systematic TB screening.

Serodiagnostic testing for active TB has been explored for the past 30 years, initially starting with evaluation of crude *M*.*tb* antigens that showed low specificity[20], followed by evaluation of purified proteins and recombinant antigens individually and in combination[21]. A recent systematic review and meta-analysis of serodiagnostic tests for active TB demonstrated high heterogeneity of test performance among studies evaluated, with sensitivities reported between 0–100% and specificities between 59–100%[8]. WHO policy recommendations on the use of serologic testing for active TB have been unfavorable due to the low accuracy of existing commercial assays and, in particular, the poor quality of existing studies[8, 9]. Despite these poor results, the potential utility of a low-cost, point of care serologic test has prompted additional research. These studies have been met with mixed results. Studies concluding that there is little potential for use of serologic testing for active disease often evaluated current serologic assays that use antigens of low importance (as demonstrated in this study) such as ESAT-6 and CFP-10[22], or evaluate antibody responses to only a single antigen[23]. Some antigens, such as the 85 complex, have not been included in current commercial serological assays. Because of the known heterogeneous nature of humoral responses to *M*.*tb*[24], serodiagnostic assays using limited numbers of antigens may be doomed to fail.

In contrast, our results are largely in agreement with prior studies that have evaluated multiplex serologic testing[10, 25, 26]. Antibody responses in a Brazilian cohort using a panel of 7 antigens (including Rv0934 which also had high variable importance in our study) demonstrated high sensitivity (93%) and specificity (87%) in detecting active TB when compared to TB negative controls from a non-endemic environment[26]. Similar results were shown in pilot studies of TB cohorts in Mali and Thailand when serum responses to a panel of antigens which included antigens evaluated in our study (Rv0831 and bfrB) demonstrated sensitivities between 73–90% and specificity of 100%[25]. Of the 8 antigens identified in our Ugandan cohort, five (Ag85A, Ag85C, Ag85B, Rv3881, Rv0934-P38) were also significantly different between patients with and without TB in a prior study from Pakistan[10]. A review of TB serodiagnostics showed promise for one of the antigens included in our panels, Rv0934, and showed correlation between antibody levels and sputum smear positivity, suggesting a role for serologic testing to improve early detection and reduce disease transmission[27]. These results support our conclusions and suggest potential for use of a panel of serodiagnostic assays across different epidemiological settings. The levels of antibodies to the remaining three antigens identified in the Pakistan study (Rv0054, Rv2031c-HSPX, Rv1860-mpt32) were not significantly different between patients with and without TB (p >0.3) and had low variable importance in our study. Despite these differences, both studies support that measuring antibody responses to multiple antigens may be necessary to account for heterogeneity of antibody responses and to optimize accuracy for TB screening. In addition, advanced classification algorithms such as SuperLearner can help identify the most promising combinations of antigens for further investigation. Of note, once validated, the prediction algorithm resulting from SuperLearner can be easily implemented on a smart phone or other mobile devices.

If further validated, the antigen panels identified here have strong potential to be developed into a low-cost, point-of-care assay. Such assays have revolutionized care for other infectious diseases such as HIV, syphilis, and malaria. A key difference for TB screening is that our study and others to date indicate that a test with sufficient accuracy will require quantitative measurement of antibody responses to multiple antigens, which presents both technical and analytic challenges to developing a point-of-care assay. Even if technical challenges could not be overcome in the short term, recent data suggest that serologic testing can be performed on dried blood spots. Samples could potentially be collected in the field and transported to a central testing site. Furthermore, the multiplex serology test reported here is rapid (approximately 2–3 hours) and scalable to handle anywhere from 1 to 360 samples per day, with the potential for even higher throughput and automation.

There are strengths and limitations to our study. A key strength is the use of a comprehensive analytic approach to identify and evaluate candidate antigens. Our data are likely robust as results were similar in the full and validation data sets. We also show that using an ensemble method like SuperLearner is better than using any one individual method **(S2 Table)**. There are also several limitations to this study. First, our analysis was performed in a hospitalized cohort. Although our results may not be generalized to community settings, healthcare facilities are one of the contexts in which the WHO recommends systematic screening for active TB and we included all patients with cough ≥2 weeks’ duration, regardless of whether cough was self-reported. Second, we excluded people living with HIV. Further studies are needed to validate our findings in other key populations targeted for systematic screening, including people living with HIV. Third, we did not assess for latent tuberculosis infection (LTBI). However, the goal of TB screening is to differentiate patients with a high likelihood of active TB from those without active TB, regardless of LTBI status.

## Conclusion

We have identified panels of antibody responses to three to eight antigens that show promise for TB screening. Accuracy approaches minimum recommended accuracy thresholds with as few as three antigens, and is greater than that reported for TB screening using symptoms or chest radiography[1]. If further validated, multiplex serologic testing could facilitate uptake of systematic TB screening of high-risk populations.

## Supporting information

S1 Table Sensitivity and specificity of panels consisting of top-ranked antigens. Performance characteristics in this table correspond to values presented in Fig 3.
(DOCX)

S2 Table Cross-validated risk of SuperLearner vs. best individual learner. * Antigens included in the analysis correspond to those shown in Fig 3.
(DOCX)
