# Supplementary material for: Evaluation of antibody responses to panels of M. tuberculosis antigens as a screening tool for active tuberculosis in Uganda
Source: PLoS One. 2017 Aug 2;12(8):e0180122. doi: 10.1371/journal.pone.0180122 (PMC5540581; doi:10.1371/journal.pone.0180122)
Supplement: S1 Table — Performance characteristics in this table correspond to values presented in Fig 3. (DOCX) [file pone.0180122.s001.docx]

| N | Antigen Panel | Full dataset | |  | Validation set only | |  |
| --- | --- | --- | --- | --- | --- | --- | --- |
|  |  | Sensitivity | Specificity |  | Sensitivity | Specificity | cvAUC |
|  |  | *% [95% CI]* | |  | *% [95% CI]* | | |
| 3 | Ag85B, Ag85A, Ag85C | 90.1  [89.4, 92.9] | 66  [47.9, 80.6] |  | 91.4  [87.8, 97.6] | 69.2  [26.2, 96] | 0.80 [0.74, 0.85] |
| 4 | + Rv0934-P38 | 90.1  [89.4, 92.9] | 72.2  [59.2, 83.1] |  | 91.5  [87.8, 100] | 74.1  [40.2, 95.7] | 0.81 [0.75, 0.86] |
| 5 | + Rv3881 | 90  [89.4, 92.5] | 82.3  [66.1, 93.5] |  | 91.6  [87.6, 99.1] | 83.7  [49.4, 100] | 0.85 [0.8, 0.9] |
| 6 | + BfrB | 90  [89.4, 92.5] | 84.1  [70.2, 96] |  | 91.8  [87.6, 99.1] | 85.1  [51.8, 100] | 0.85 [0.8, 0.91] |
| 7 | + Rv3873 | 90.2  [89.4, 92.9] | 86.8  [72.6, 96] |  | 92.1  [87.6, 100] | 86.6  [51.6, 100] | 0.87 [0.82, 0.92] |
| 8 | + Rv2878c | 90.6 [89.4, 93.8] | 88.6  [78.2, 97.6] |  | 92.8  [87.8, 100] | 88.4  [65.9, 100] | 0.9 [0.86, 0.94] |
| 9 | + Rv3507 | 90.6  [89.4, 93.8] | 88.4  [77.4, 96.4] |  | 92.9  [87.8, 100] | 88.4  [62.5, 100] | 0.9 [0.86, 0.94] |

**S1 Table. Sensitivity and specificity of panels consisting of top-ranked antigens.**
