# Supplementary material for: Evaluation of antibody responses to panels of M. tuberculosis antigens as a screening tool for active tuberculosis in Uganda
Source: PLoS One. 2017 Aug 2;12(8):e0180122. doi: 10.1371/journal.pone.0180122 (PMC5540581; doi:10.1371/journal.pone.0180122)
Supplement: S2 Table — * Antigens included in the analysis correspond to those shown in Fig 3. (DOCX) [file pone.0180122.s002.docx]

|  | Mean (standard error) | |
| --- | --- | --- |
| Panel size* | SuperLearner | Best Individual Learner |
| 3 | 0.173 (0.014) | 0.171 (0.013) |
| 4 | 0.163 (0.013) | 0.163 (0.013) |
| 5 | 0.141 (0.014) | 0.14 (0.015) |
| 6 | 0.13 (0.014) | 0.13 (0.014) |
| 7 | 0.133 (0.014) | 0.133 (0.015) |
| 8 | 0.129 (0.013) | 0.129 (0.013) |
| 9 | 0.123 (0.013) | 0.123 (0.013) |

**S2 Table. Cross-validated risk of SuperLearner vs. best individual learner** .

* Antigens included in the analysis correspond to those shown in Figure 1 and S1 Table.
